# Beneficial Effects of Laurel (*Laurus nobilis* L.) and Myrtle (*Myrtus communis* L.) Extract on Rat Health

**DOI:** 10.3390/molecules27020581

**Published:** 2022-01-17

**Authors:** Marija Berendika, Sandra Domjanić Drozdek, Dyana Odeh, Nada Oršolić, Petar Dragičević, Marijana Sokolović, Ivona Elez Garofulić, Domagoj Đikić, Irena Landeka Jurčević

**Affiliations:** 1 Department of Food Quality Control, Faculty of Food Technology and Biotechnology, University of Zagreb, Pierottijeva 6, 10000 Zagreb, Croatia; mberendika@veinst.hr (M.B.); sandra.domjanic-drozdek@zvu.hr (S.D.D.); ielez@pbf.hr (I.E.G.); ilandeka@pbf.hr (I.L.J.); 2 Department of Animal Physiology, Faculty of Science, University of Zagreb, Rooseveltov trg 6, 10000 Zagreb, Croatia; dyana.odeh@biol.pmf.hr (D.O.); domagoj.djikic@biol.pmf.hr (D.Đ.); 3 School of Medicine, University of Zagreb, Šalata 3, 10000 Zagreb, Croatia; pd4082@gmail.com; 4 Croatian Veterinary Institute Zagreb, Savska Cesta 143, 10000 Zagreb, Croatia; m_sokolovic@veinst.hr

**Keywords:** Laurel extract, Myrtle extract, gut microbial enzyme activity, antioxidative activity in tissue, probiotic bacteria

## Abstract

Polyphenols of Laurel and Myrtle exhibit structural diversity, which affects bioavailability, metabolism, and bioactivity. The gut microbiota plays a key role in modulating the production, bioavailability and, thus the biological activities of phenolic metabolites, particularly after the intake of food containing high-molecular-weight polyphenols. The aim of this study was to investigate whether the polyphenolic components of Laurel and Myrtle aqueous extract have beneficial effects on rat health. The growth of lactic acid bacteria (LAB), β-glucuronidase, β-glucosidase, β-galactosidase activity, pH value, body weight change and food efficacy ratio after intragastric treatment of rats with Laurel and Myrtle extract at doses of 50 and 100 mg/kg for two weeks were investigated. The endogenous populations of colonic probiotic bacteria (Lactobacilli and Bifidobacteria) were counted on selective media. According to the obtained data, Laurel extract in the applied dose of 50 and 100 and Myrtle extract (100 mg/kg) positively affects the rats health by increasing the number of colonies of Lactobacilli and Bifidobacteria compared to the control group, causes changes in glycolytic enzymatic activity and minor change in antioxidative tissue activity. In addition, high doses of Laurel increase food efficiency ratio, while Myrtle has the same effect at a lower dose.

## 1. Introduction

In recent years, the need for the use of healthy food in the prevention and treatment of diseases in a “natural” way, with proven scientific effectiveness, has been increasing worldwide. A large number of medicinal plants, such as Myrtle (*Myrtus communis* L., fam. Myrtaceae) and Laurel (*Laurus nobilis* L., fam. Lauraceae) are a source of new compounds that have antioxidant, antibacterial, antiviral, antifungal, anti-inflammatory and immunomodulatory activity [1,2,3,4]. These aromatic spices and herbs can be used in culinary, food, perfume and cosmetic industries, as well as for medicinal purposes (antimicrobial and antioxidative agents). Generally, in folk medicine, a decoction of leaves and fruits is used orally for the treatment of stomach aches, hypoglycaemia, dysbiosis, cough, constipation, poor appetite, as well as for external application for wound healing [5,6]. In folk medicine in some countries such as Turkey, Italy, Sardinia and other countries in the Mediterranean region and Europe, the fruits and leaves of these plants are used in the treatment of many types of infectious diseases, including diarrhoea and dysentery. The leaves of these plants are used as antiseptic and antiinflammatory agents, as well as a mouth wash for the treatment of candidiasis [1,2,3,4,5,6] or are applied on the skin for the treatment of dermatitis. Thus, Laurus leaves are rich in the needed trace elements and tannins that help in the elimination of body toxins [7] and it has been traditionally used orally to treat flatulence, digestive problems, nerve pain, and epilepsy [3,6,7]. It has been shown that infusions made from the Myrtus leaves are used as stimulants, antiseptics, astringents and hypoglycaemics, and they are considered to be a health remedy for asthma, eczema, psoriasis, diarrhoea, gastrointestinal disorders and urinary infections. The activity of these plants is based on their components such as phenolic acids, flavonoids, alkaloids, and simple phenols, lignans, carotenoids, vitamins, terpenoids, [1,2,3,5,6]. The leaves of these plants contain substances such as tannins, coumarins, galloyl glucosides, caffeic acid, gallic acid, ellagic acid, and various terpenoid compounds [8].

Polyphenols may act on gut microbiota to favour the increase in beneficial bacteria such as *Lactobacillus* spp. and *Bifidobacterium* spp. and hamper the increase in pathogenic bacteria such as *Clostridium* spp. In addition, the gut microbiota is able to metabolize polyphenols, making them more bioactive, and more easily absorbed than the original compounds [9,10,11]. Polyphenols can also help to control body weight by inhibiting appetite, improving lipid metabolism, and inhibiting pancreatic lipase activity [9,10,11,12]. On the other hand, herbs contain active substances that can improve digestion and metabolism and possess bacterial and immunostimulant action of animals [9,10,11,12,13]. In addition, a diet with medicinal plants can induce the promotion of growth performance [14], increase stress tolerance [15], and enhance immune system efficiency [13,14,15]. Both polyphenols and microbiota metabolites may act on metabolic pathways and confer health benefits [9,10,11,12,13]. In addition, phenolic compounds are metabolized as “typical xenobiotics” by the human body, and such metabolism can influence their antioxidant and prooxidant abilities that can result in oxidative stress of different intensity with respective consequences [16].

Despite the possible benefits of polyphenols for human health through modulation of the microbiome, and its protection of the mucous membranes of the respiratory, digestive and urogenital systems, studies about *Lactobacillus* and *Bifidobacterium* and their enzymatic activity in weight regulation and antioxidative capacity of tissue are scarce. Thus, the aim of this study was to investigate whether the intake of polyphenolic components of Laurel and Myrtle extract has beneficial effects on rat health. Based on the above, we assessed the effect of Laurel and Myrtle extract on the growth of lactic acid bacteria (LAB), β-glucuronidase, β-glucosidase, β-galactosidase activity, pH value, body weight change and food efficacy ratio after intragastric treatment of rats with Laurel and Myrtle aqueous extract at doses of 50 and 100 mg/kg for two weeks.

The antioxidant capacity of liver and kidney tissue was tested with different antioxidant mechanisms using a number of assays such as Ferric reducing antioxidant power (FRAP) assay, ABTS assay (2,2′-azino-bis(3-ethylbenzothiazoline-6-sulfonic acid), and DPPH• free radical scavenging assay (2,2-Diphenyl-1-(2,4,6-trinitrophenyl) hydrazyl. In addition, an analysis of oxidative stress parameters such as malondialdehyde (MDA, biomarkers for lipid peroxidation), glutathione level (GSH, the most abundant thiol in animal cells) protein carbonyl content (as a marker of damage) and catalase activity (a ubiquitous antioxidant enzyme that degrades hydrogen peroxide into water and oxygen) was done to assess (i) the usefulness of Laurel and Myrtle extract as antioxidants, (ii) differences between tissues and (iii) the underlying mechanism of any potential antioxidant capacity in protecting the cell from oxidative damage caused by reactive oxygen species (ROS).

## 2. Results

### 2.1. Total Phenolic Compound, UPLC-MS^2^ Analysis

The total amounts of phenol were estimated by the Folin–Ciocalteu method for each extract and were expressed in mg of gallic acid equivalents (GAE)/100 g of sample. Spectrophotometric analysis has shown that total polyphenol content in Laurel and Myrtle extract was 51.36 mg/mL and 48.64 mg/mL, respectively. The main polyphenols in both extracts obtained by using the UPLC-MS^2^ are shown in Table 1. The highest percentage of polyphenolic components in Laurel extract belongs to caffeic acid (19.31%), apigenin (12.63%), Quercetin-3-rutinoside (12.24%), Quercetin-3-glucoside (10.74%), Epicatechin (6.77%), Kaempferol-3-*O*-hexoside (5.24%) and lsorhamnetin-3-hexoside (4.66%) while in Myrtle extract, the main polyphenolic components are Myricetin-3-*O*-rham (36.68%), Myricetin-3-*O*-gallac (33.20%), Miricetin (14.48%) and 5-*O*-galloyl quinic Acid (7.96%).

### 2.2. Assessment of the Effects of Laurel and Myrtle Extract on Weight Gain and Food Efficiency Ratio

**Figure 1:**
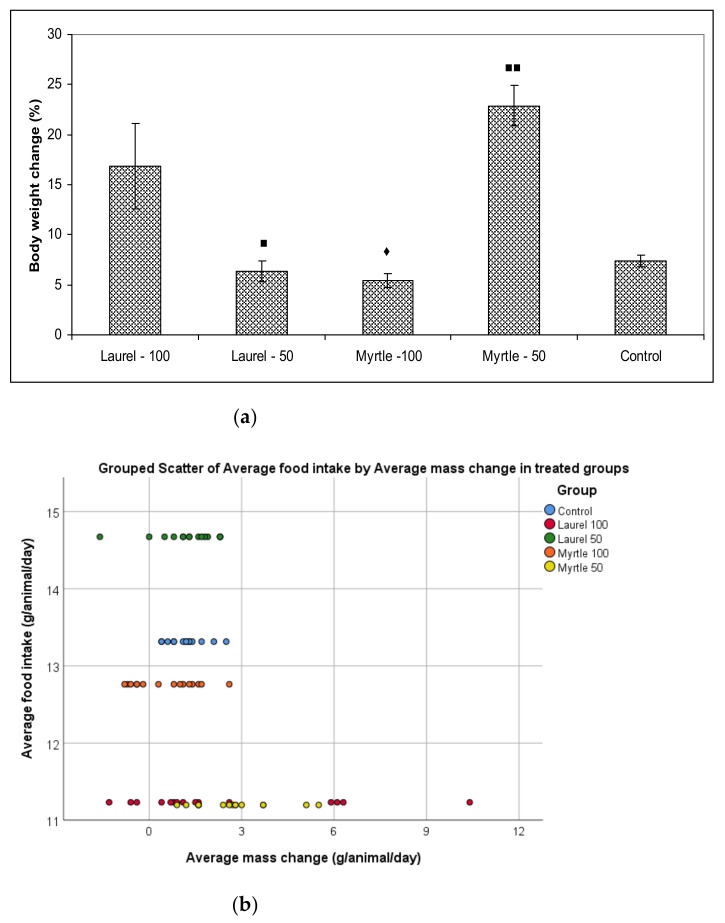
The effect of Laurel and Myrtle extract on body weight change during the experiment (**a**) and the scattergram of correlative relationship between average food intake and mass change of animals per day (**b**). Male rats (*n* = 5) were administered Laurel and Myrtle extracts *ig* at a dose of 50 and 100 mg/kg once a day for 14 days. The control group was treated with *ig* saline. The results are expressed as the mean value of each experimental group ± SE of the mean of two different observations. ^■^ Significantly different in relation to Myrtle-100 (^■^ *p* < 0.05; ^■■^ *p* < 0.01). ^♦^ Significantly different in relation to Laurel-100 (^♦^ *p* < 0.05).

It is known that medicinal plants in the diet of animals can contribute to the feeding strategy and consequently its growth performance through their antioxidant, anti-inflammatory, anti-microbial, and antidiarrheal effects [14,17]. Thus, food intake, weight gain, and food efficiency ratio in the experimental groups is shown in Table 2. Intragastric administration of Laurel or Myrtle at a dose of 50 or 100 mg/kg did not cause significant changes in food or water intake. A significant increase in daily animal weight was observed after the intragastric application of Laurel at a dose of 100 mg/kg and Myrtle at a dose of 50 mg/kg as compared to Myrtle-100 (*p* < 0.05 and 0.01). The Laurel-100 and Myrtle-50 groups of rats had a significantly greater food efficiency ratio (FER) in relation to Myrtle-100 (*p* < 0.05 and 0.01) but without significance compared to the control group (0.25 ± 0.03 and 0.32 ± 0.02 versus 0.12 ± 0.01, respectively). The percentage (%) of weight change is shown in Figure 1a. The sequence of weight change (%) from largest to smallest is shown as follows: Myrtle-50 (22.89%); Laurel-100 (16.82%); Control (7.41%); Laurel-50 (6.38%); and Myrtle-100 (5.39%).

The correlation between body weight change and food intake/food efficiency ratio is shown in Figure 1b. The correlation within the group could not be detected. The scattergram shows that the Laurel-50 group had a higher average daily food intake than the control but did not have an increase in body mass. Myrtle-50 and Laurel-100 showed decreased food intakes but the body mass was retained similar to the control or even increased in some individuals. It seems that individual differences within the group may affect the different absorption of phenolic components and their effect in the body.

### 2.3. Assessment of the Effects of Laurel and Myrtle Extract on Probiotic Bacteria

It is known that biological activity of polyphenols is significantly different in in vitro and in vivo conditions. According to Bayliak et al. [16] the biological activity of polyphenols and their secondary metabolites depends on pH and enzyme availability. In addition, phenolic compounds are not metabolized as “typical xenobiotics” by the human body, and as such the metabolism can influence their antioxidant and prooxidant abilities as well as have different consequences on the human body. Accordingly, the relationship between polyphenol levels, antioxidant activity, and lactic acid levels, pH, and enzymatic activity of intestinal contents was investigated.

It can be seen in Supplementary Materials (Table S1) that there are no significant changes in the pH value of the intestinal contents of the colon in rat.

Polyphenols are known to exhibit growth-promoting effects, namely prebiotic actions, on intestinal bacteria and selective antimicrobial activity against pathogenic bacteria. Weight gain is most commonly associated with changes in the intestinal microbiota, especially *Lactobacillus* and *Bifidobacterium*, which are most commonly used in agriculture. Moreover, some data suggest that *Lactobacillus* containing probiotics may affect weight regulation in humans and animals [18] while some strains of *Bifidobacterium* genus are believed to support the lean status [19,20,21,22,23]. In line with this, we analysed the effect of Myrtle and Laurel extract on the number of Lactobacillus and *Bifidobacterium* colonies and the enzymatic activity of the intestinal microbiota.

**Figure 2:**
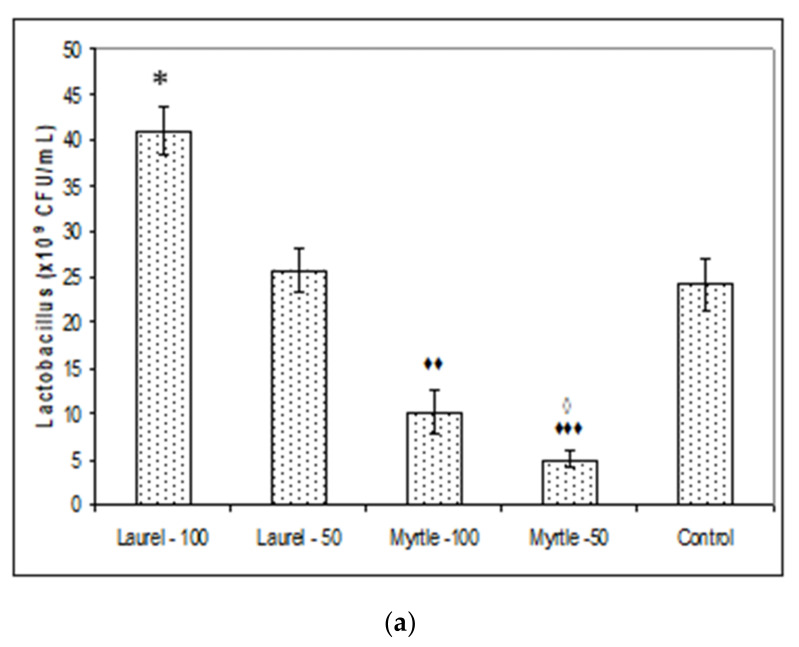

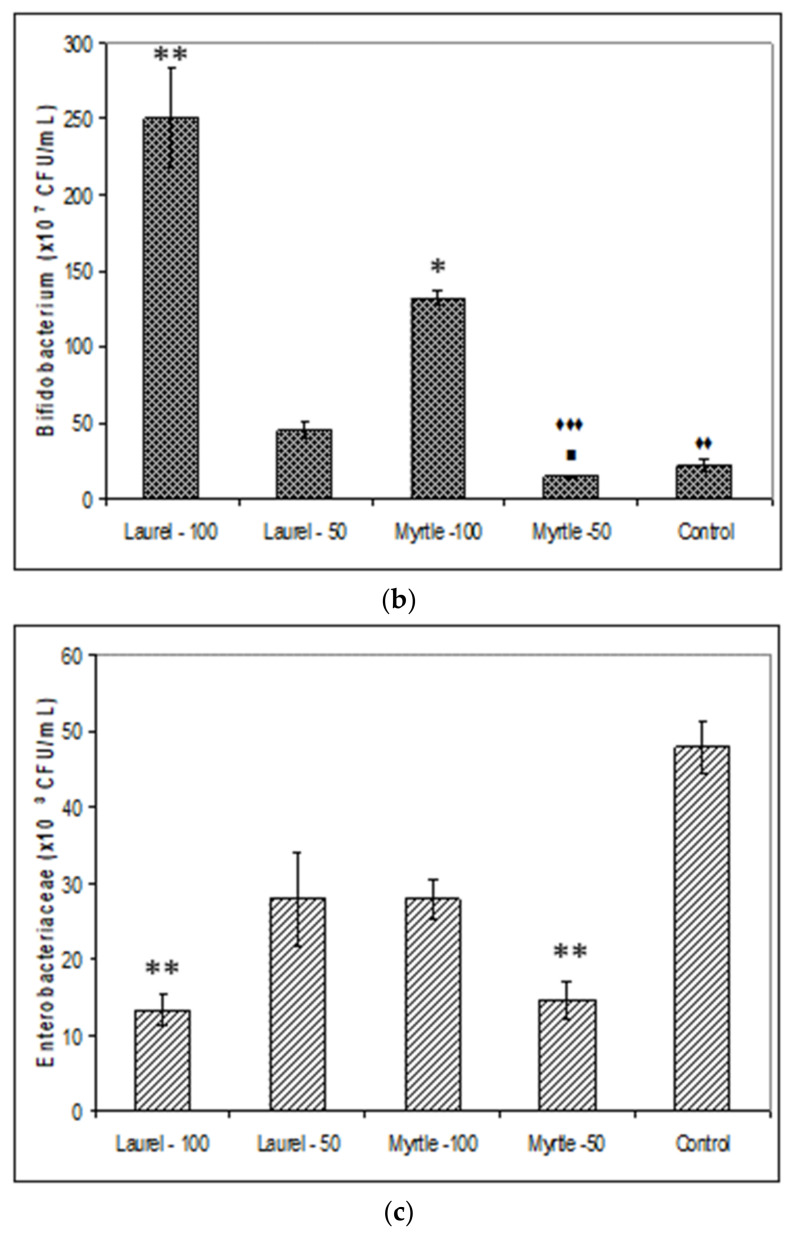
The effect of Laurel and Myrtle extract on the number of colonies of *Lactobacillus* (**a**), *Bifidobacterium* (**b**) and *Enterobacteriaceae* (**c**) formed on selective media. Male rats (*n* = 5) were administered Laurel and Myrtle extracts *ig* at a dose of 50 and 100 mg/kg once a day for 14 days. The control group was treated with *ig* saline. The results are expressed as the mean value of each experimental group ± SE of the mean of two different observations. * Significantly different in relation to the control (* *p* < 0.05; ** *p* < 0.01). ^♦^ Significantly different in relation to Laurel-100 (^♦♦^ *p* < 0.01; ^♦♦♦^ *p* < 0.001). ^■^ Significantly different in relation to Myrtle-100 (^■^ *p* < 0.05). ^◊^ Significantly different in relation to Laurel-50 (^◊^ *p* < 0.05).

Analysis of the number of colonies of probiotic bacteria showed that the intake of Laurel extract (100 mg/kg) increased the number of *Lactobacillus* (*p* < 0.05) in relation to the control by about two times (Figure 2a). It also increased the number of *Bifidobacterium* colonies compared to the control group (*p* < 0.01). The number of colonies of *Bifidobacteria* at same dose of Laurel was (45.00 ± 5.47 and 250 ± 32.86 vs. 21.66 ± 4.038 CFU/mL) in comparison to the control (Figure 2b). Myrtle at a dose of 50 mg/kg showed a lower number of *Lactobacillus* and *Bifidobacterium* colonies compared to the control, while the number of Bifidobacterium at a dose of 100 was significantly higher than the control group (*p* < 0.05). Statistical significance between different treatments of rats with Laurel or Myrtle extract for the number of *Lactobacillus* colonies (Figure 2a) exists between the following groups: Laurel-100 vs. Myrtle-100 (*p* < 0.01); Laurel-100 vs. Myrtle-50 (*p* < 0.001) and Laurel-50 vs. Myrtle-50 (*p* < 0.05). The number of *Bifidobacterium* colonies between different treatments of rats with Laurel or Myrtle extract was statistically significant between the following groups: Laurel-100 vs. Myrtle-50 (*p* < 0.001) and Myrtle-100 vs. and Myrtle-50 (Figure 2b). Significant reduction of *Enterobacteriaceae* was observed in Laurel-100 and Myrtle-50 groups compared to the control; treatment with Laurel-100 reduced the number of the colony by 72% (*p* < 0.01), Myrtle-50 by 69.57% (*p* < 0.01) while Laurel-50 and Myrtle-100 show a reduction of 41.90% without significance (Figure 2c).

Biochemical confirmation of *Lactobacillus* species was performed using API 50 CHL. The data of biochemical identification of Laurel supplement at dose 50 confirmed the presence of *Lactobacillus fermentum* (99.9%) and Laurel-100 *Lactobacillus fermentum* (72.2%) while treatment with Myrtle 50 confirmed—*Lactococcus lactis* ssp lactis 1 (76.8%) and *Lactococcus raffinolactis* (49.4%) while Myrtle at a dose of 100 confirmed the presence of *Lactobacillus plantarum* 1 (88.2%).

**Figure 3:**
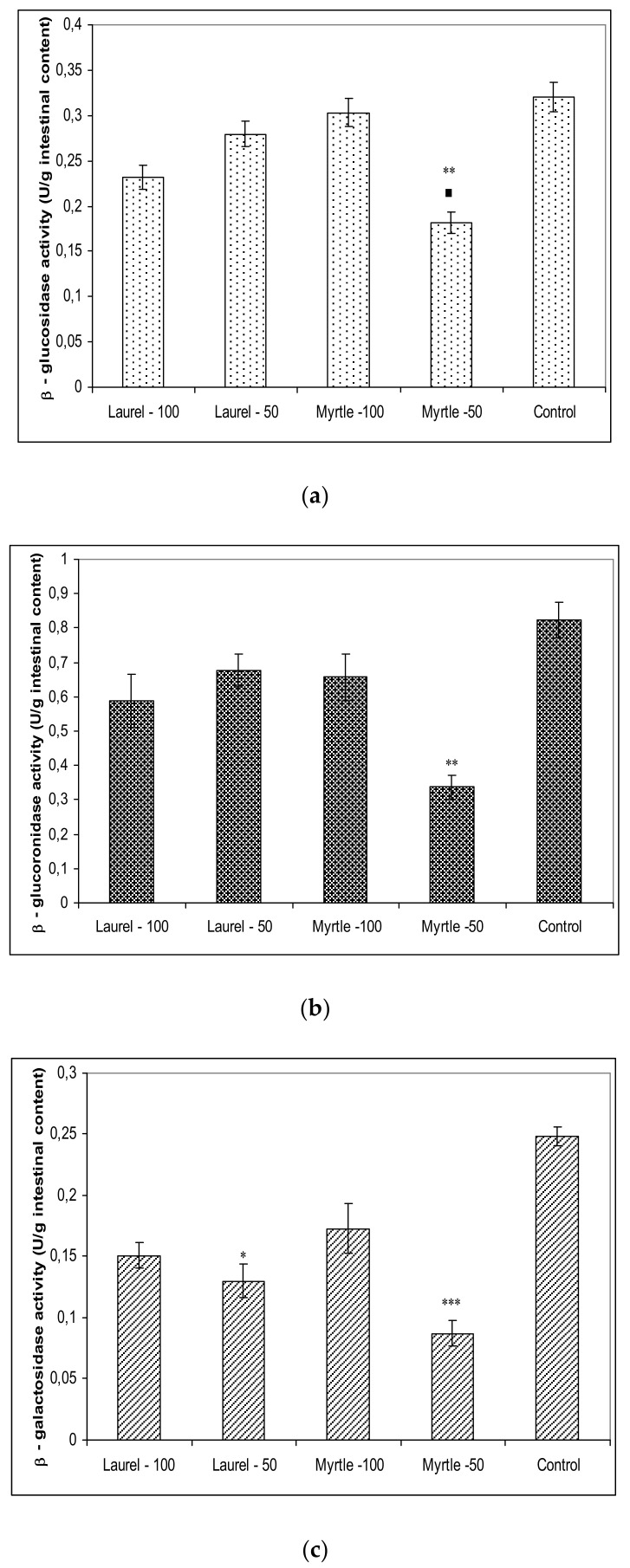
The effect of Laurel and Myrtle extract on faecal bacterial enzymes β-glucosidase (**a**), β-glucuronidase (**b**), β-galactosidase (**c**) activity. Male rats (*n* = 5) were administered Laurel and Myrtle extracts *ig* at a dose of 50 and 100 mg/kg once a day for 14 days. The control group was treated with *ig* saline. The results are expressed as the mean value of each experimental group ± SE of the mean of two different observations. * Significantly different in relation to control (* *p* < 0.05; ** *p* < 0.01; *** *p* < 0.001). ^■^ Significantly different in relation to Myrtle-100 (^■^ *p* < 0.05).

Figure 3 summarises the activities of β-glucosidase, β-glucuronidase, β-galactosidase in fresh faecal samples. The activities of these enzymes were increased in the control group rats compared to the Laurel and Myrtle treated rats. The percentage reduction of β-glucosidase activity (U/g intestinal content) compared to the control group was as follows: Myrtle-50 (43.30%); Laurel-100 (27.66%); Laurel-50 (12.66%); and Myrtle-100 (5.22%). The reduction in β-glucuronidase activity was: Myrtle-50 (58.88%); Laurel-100 (28.15%); Myrtle-100 (19.90%) and Laurel-50 (17.66%). A similar sequence was seen in reduction of β-galactosidase activity: Myrtle-50 (65.61%); Laurel-50 (47.97%); Laurel-100 (39.74%); and Myrtle-100 (30.81%).

### 2.4. Antioxidative Capacity and Oxidative Stress Biomarkers of Liver and Kidney

Some research has shown that the consumption of oxidized oils in food can lead to the induction of oxidative stress in the host [24]. Based on the above, it is important that both the food and the living organism be protected from excessive levels of oxidative stress. In this study, we investigated the effect of Myrtle and Laurel as a food supplement and their ability to alter the antioxidant capacity of the kidneys and liver through their antioxidant activity as well as their ability to change probiotic bacteria and their enzymatic activity.

**Figure 4:**
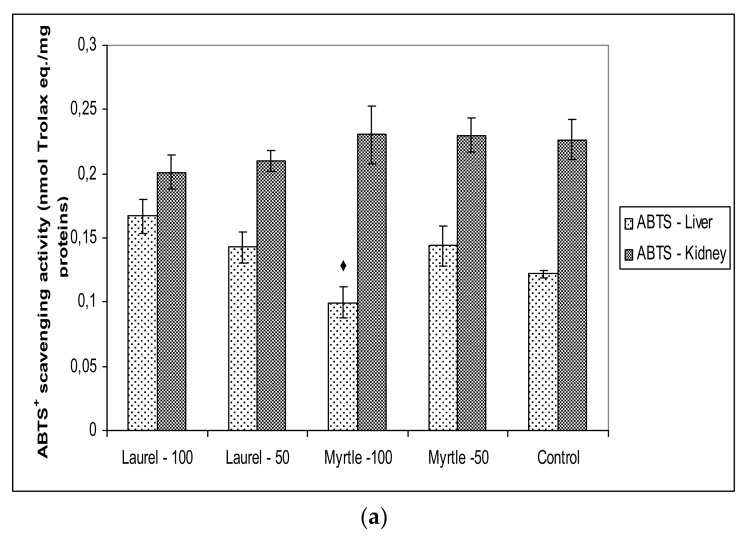

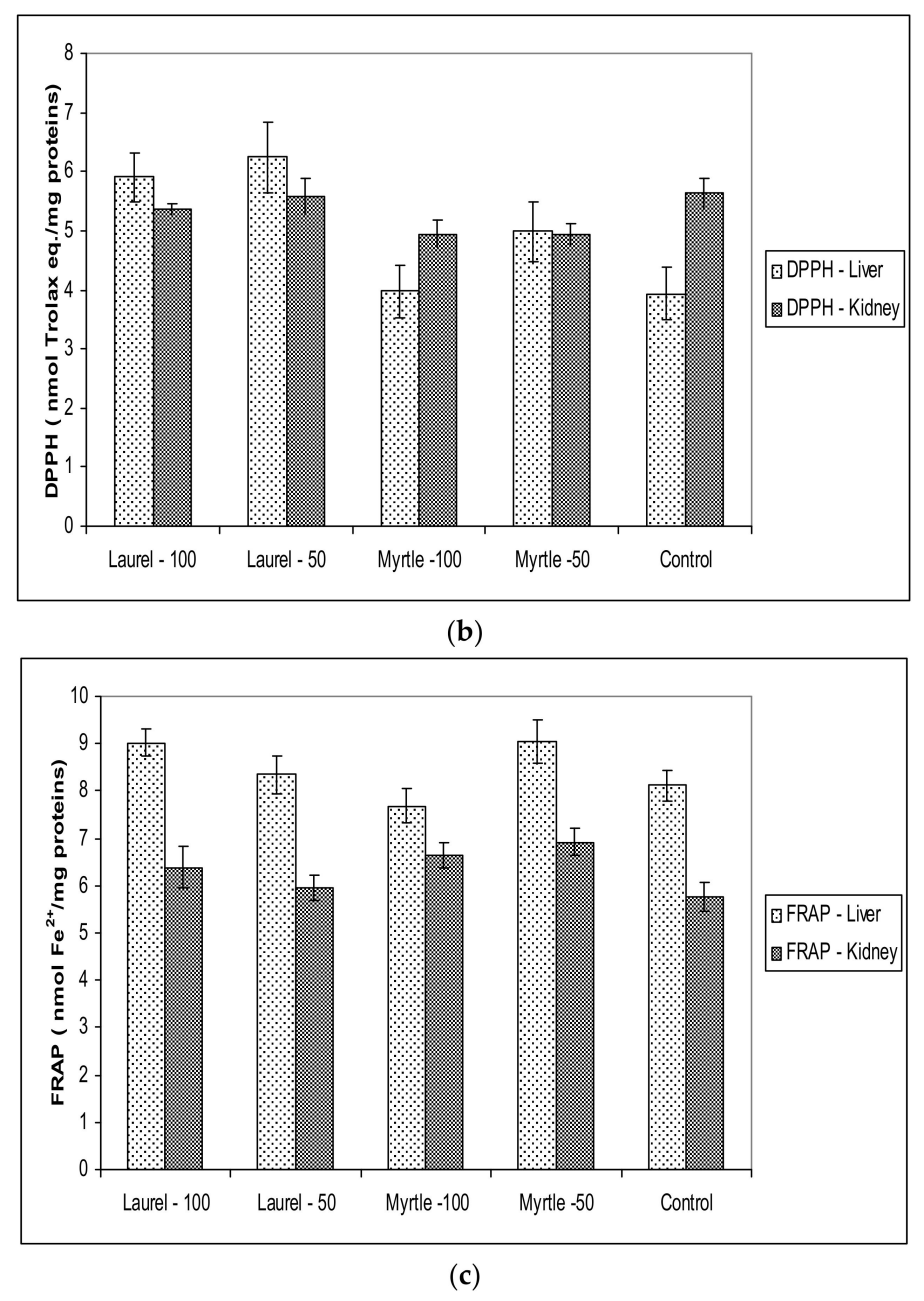
The effect of Laurel and Myrtle extract on the antioxidative capacity of the liver and kidney tissues homogenates measured by ABTS (**a**), DPPH (**b**) and FRAP (**c**) activity. Male rats (*n* = 5) were administered Laurel and Myrtle extracts *ig* at a dose of 50 and 100 mg/kg once a day for 14 days. The control group was treated *ig* with saline. The results are expressed as the mean value of each experimental group ± SE of the mean of two different observations. ^♦^ Significantly different in relation to Laurel-100 (^♦^ *p* < 0.05).

**Figure 5:**
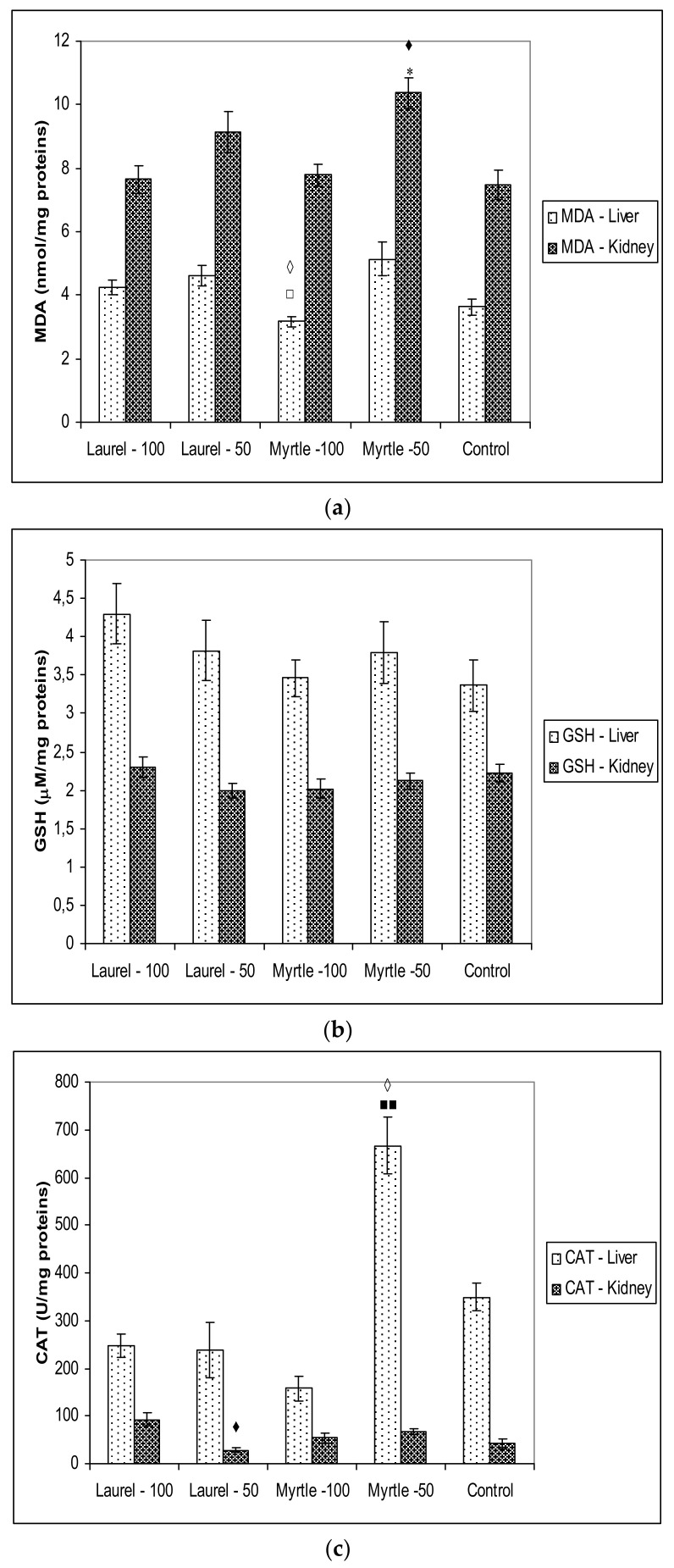

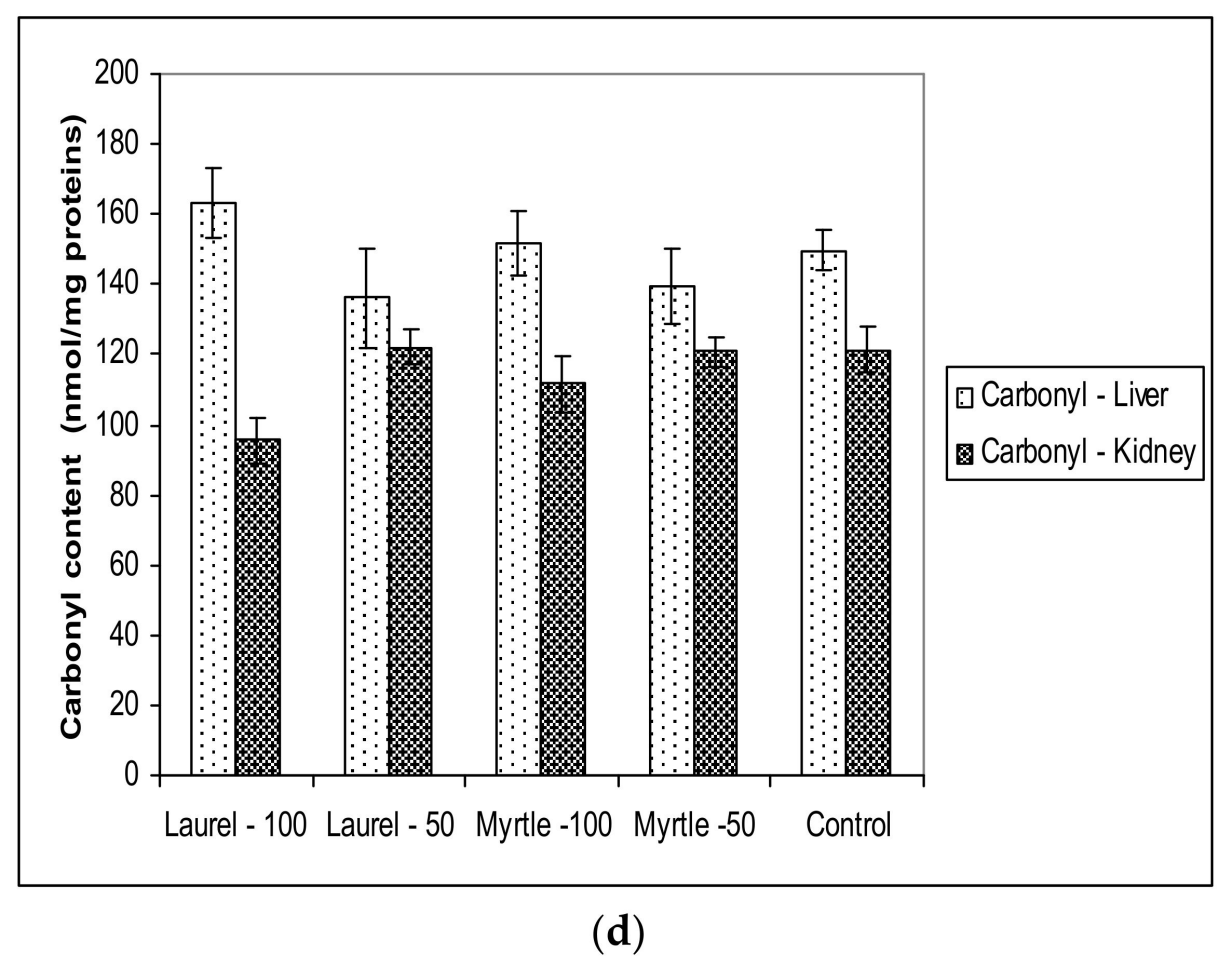
The effect of Laurel and Myrtle extract on oxidative stress biomarkers in the liver and kidney tissues homogenates measured by MDA level (**a**), GSH level (**b**) CAT activity (**c**) and carbonyl content (**d**). Male rats (*n* = 5) were administered Laurel and Myrtle extracts *ig* at a dose of 50 and 100 mg/kg once a day for 14 days. The control group was treated with *ig* saline. The results are expressed as the mean value of each experimental group ± SE of the mean of two different observations. * Significantly different in relation to Control (* *p* < 0.05). ^■^ Significantly different in relation to Myrtle-100 (^■■^ *p* < 0.01); ^♦^ Significantly different in relation to Laurel-100 (^♦^ *p* < 0.05) ^□^ Significantly different in relation to Myrtle-50 (^□^ *p* < 0.05); ^◊^ Significantly different in relation to Laurel-50 (^◊^ *p* < 0.05).

The antioxidant capacities and antioxidative protective molecules in rat tissues assayed by FRAP, ABTS and DPPH, MDA, protein carbonyl content, GSH and CAT activity (Figure 4 and Figure 5).

The radical scavenging capacities were assessed with the lipid soluble DPPH radical as well as the water soluble ABTS radical while the compounds that chelate metals such as Fe are considered to have antioxidant capacity and assessing “antioxidant power”. Interestingly, we did not obtain statistically significant differences in antioxidant capacity between the groups treated with Laurel and Myrtle extracts and controls, except between the rats treated with Myrtle-100 and Laurel-100 (*p* < 0.05) in liver tissue (Figure 4).

For oxidative stress biomarkers, treatment of rats with Myrtle-50 induced a significant increase in the level of MDA in the kidney compared to Laurel-100 (*p* < 0.05) and control (*p* < 0.05) while the same dose of Myrtle in the liver (50 mg/kg) increased CAT activity compared to the rats treated with Laurel-50 (*p* < 0.05) and Myrtle-100 (*p* < 0.01). No changes were observed with the amounts of GSH and carbonyl content in the liver and kidney after treatment with Laurel and Myrtle extract (Figure 5).

## 3. Discussion

There is an increased interest in following a healthy lifestyle and consuming fruits, vegetables and the use of medicinal herbs, rich in polyphenols, because of their benefits to the human body. Food products enriched with various forms of medical plants are sources of pro-health components. Nevertheless, in many cases, the level of their activities is changed in in vivo conditions and their beneficial effect depends on the applied diet and its effect on the intestinal microbiota and their enzymatic activity. In this study we investigated the physiological indices of laboratory rats as a response to diets supplemented with aqueous extracts of medicinal plants such as Laurel and Myrtle and their ability to alter the antioxidant capacity of the kidneys and liver through their antioxidant properties as well as their ability to increase probiotic bacteria and their enzymatic activity.

Two weeks of administration of diets supplemented with Laurel and Myrtle extract did not cause differences in the daily diet intake but induced changes in body weight gains and FER of rats in the Myrtle-50 group compared to the control and Myrtle-100 groups (Table 2). In addition, body weight gain was increased when rats were given a diet with a Laurel extract at a dose of 100 mg/kg compared to Myrtle-100. The observed decrease in the weight of rats in Myrtle-100 group may be explained by increased levels of Myricetin, Myricetin 3-*O*-galactoside and Myricetin 3-*O*-rhamnoside (Table 1) as active molecules with pharmaceutical potentials present in Myrtle-100 treatment. It has been demonstrated that Myricetin administration attenuated increases in glucose, total triglyceride, and cholesterol levels and affected the levels of leptin and adiponectin [25,26]. In addition, tannins can contribute to this. Tannins (or tannoids) such as 5-*O*-galloylquinic acid are a class of astringent, polyphenolic biomolecules that bind to and precipitate proteins and various other organic compounds including amino acids and alkaloids. Generally, tannins induce a negative response when consumed [1,2]. Since we did not notice a difference in food intake, we conclude that the appetites of the rats were unaffected by increased myricetin level present in Myrtle-100 treatment.

Furthermore, a large amount of the literature data is consistent with our data (Figure 3) that the addition of polyphenols can affect colon ecosystems and their enzymatic activity [13,18,19,20,21,22,23,25]. One of their effects was demonstrated as the lowering of the microbiological activities of β-glucuronidase, β-galactosidase and β-glucosidase activities after treatment with Myrtle at a dose of 50 mg/kg. The animal gut microbiota, similar to the human, represents a complex and dynamic microbial community in the gastrointestinal tract which co-evolves and co-develops with its host [26]. Thus, gut bacterial β-glucuronidases catalyse the removal of glucuronic acid from liver-produced β-glucuronides. Glucuronidation is a major detoxification pathway in the mammalian liver, where the activities of these enzymes affect the physiological activities and toxicities of various xenobiotics and drugs increasing the lifetimes of these compounds in the circulation. These reactions can have deleterious consequences when they reverse xenobiotic metabolism. According to Dashnyam [27] two broad functional groups of β-glucuronidases can be categorized in accordance with structure-function: (i) β-glucuronidases of opportunistic bacteria as the major contributors to xenobiotic-induced toxicity in the gut; (ii) commensal bacteria such as *Lactobacillus* spp. and *Bifidobacterium* spp. for maintaining a healthy level of gut bacterial β-glucuronidases. In addition to being involved in the toxicity of carcinogenic aromatic compounds, these enzymes have also been essential for the recycling of important endogenous molecules and for the regeneration of beneficial natural products [27,28,29] since their biological activity is greater than their corresponding glucuronides [27]. It seems that Laurel increases the abundance of *Lactobacillus* and *Bifidobacterium* (Figure 2) and thus reduces the abundance of opportunistic bacteria [30]. Thus, β-glucuronidases activities in our study are responsible for maintaining a healthy interaction between the host and gut microbiota while reduced levels of this enzyme by Laurel or Myrtle may be important as dietary protection compounds against tumour incidence by chemical and natural carcinogens. Laurel at both doses may reinforce the growth of Lactobacilli in the intestine improving overall health and nutritional elements readiness and performance in rat, possible by high amount of minerals and that are related to the effectiveness of enzymatic systems in the liver and as a source of antioxidants such as polyphenols/flavonoids components [3,4,6]. It seems that the increased number of Lactobacilli colony (Figure 2a) may be one of the reasons for increased weight gain. According to results of Million et al. [19] where authors demonstrated that *L. acidophilus*, *L. ingluviei* or *L. fermentum* resulted in weight gain whereas specific strains of *L. gasseri* and *L. plantarum* used as food supplements demonstrated an anti-obesity effect. Indeed, different *Lactobacillus* species are associated with different effects on weight change that are host-specific. Using the API 50 CHL test, we confirmed that the administration of Laurel extract at a dose of 50 mg/kg showed the presence of *Lactobacillus fermentum* (99.9%) colonies and Laurel-100 *Lactobacillus fermentum* (72.2%). According to Drissi et al. [31] *Lactobacillus* spp. associated with genomes of weight gain can encode ubiquitous enzymes important in the β-oxidation pathway of fatty acid degradation and various biosynthetic pathways and thus might be able to mobilize energy and carbon stored in fatty acids through β-oxidation. Further studies are needed to clarify the role of *Lactobacillus* species in the human energy harvest and weight regulation. Polyphenols/flavonoids compounds, including kaempferol, luteolin, apigenin, quercetin, caffeic acid and proanthocyanidin act as vital precursors to increase the numbers of beneficial bacteria such as *Lactobacillus* and taking advantage of the products of these beneficial bacteria, such as lactic acid, which is the source of energy for intestinal cells and increases their activity, absorption and divisions and thus improve general health of the rats. In addition, an increased number of *Lactobacillus* colonies may exert their protective or therapeutic effect through the production of metabolites such as lactic acid, hydrogen peroxide (H_2_O_2_) and antimicrobial compounds such as bacteriocin, reduction of gut pH by stimulating the lactic acid producing microflora, competition for binding of receptor sites that pathogens occupy, stimulation of immunomodulatory cells and competition with pathogens for available nutrients [9,10,11,12,13,29]. Some of the benefits of *Lactobacillus* in the maintenance of the intestinal microbial ecosystem is seen in growth enhancement of farm animals, protection from pathogens, alleviation of lactose intolerance, relief of constipation, anticholesterolaemic effect and immunostimulation [9,10,11,12,13,18,19,20,21,29,30]. Thus, a strong reduction in abundance of *Enterobacteriaceae* can also contribute to growth promotion and health performance of rats after ingestion of Laurel extract at higher dose (Figure 2c).

On the other hand, the addition of Mirta at a dose of 50 mg/kg, leads to the best weight gain, which is partly attributed to changes in the activity of metabolic enzymes such as glucuronidase and glucosidase [32,33]. Some authors demonstrated significantly higher β-glucuronidase activity in humans and animals that had lost weight. These data are consistent with our finding that a decrease in enzymes leads to weight gain, which is consistent with data from a recent study that reported an increase in β-glucuronidase activity in obese volunteers following a weight-loss diet. [32]. In addition to glucuronidase and glucosidase activity, biochemical confirmation of *Lactobacillus* species by API 50 CHL test confirmed the presence of *Lactococcus lactis* ssp lactis 1 (76.8%) and *Lactococcus raffinolactis* (49.4%) colonies [34,35]. These probiotic strains have a number of genes, which code diverse enzymes to help many important metabolic pathways such as the urea cycle related to the metabolism, taurine biosynthesis, and de novo synthesis of nucleotides in the intestine, enhancing feed efficiency and weight gain (Table 2, Figure 1). Interestingly, Myrtle at a dose of 100 confirmed the presence of *Lactobacillus plantarum* 1 (88.2%) which was linked with weight loss (Figure 1). According to our data, it seems that different doses might have different effects; a dose at 100 mg/kg of Myrtle induces weight loss but a dose of 50 mg/kg can be linked to growth promotion. Based on the above, the aim of our further research will be to explain this association of glycolytic enzyme activity with microbial diversity and their effect in relation to dose on body weight.

According to the literature data [36,37], β-glucosidases can exert either beneficial or harmful effects, as they form aglycones from a range of different plant glucosides, which might exhibit either toxic/mutagenic or health-promoting effects. These enzymes, release a wide range of plant secondary metabolites from their β-d-glucosylated precursors, increasing the bioavailability of health-promoting, antioxidative plant metabolites. On the other hand, adverse health effects caused by increased bioavailability of dietary toxins and xenobiotics were also reported.

Moreover, β-galactosidase is also a member of the glycosyl hydrolysis enzyme, which may cause the release of glucose and galactose by way of cleavage of the β-1,4-D-galactosidic linkage of lactose [38]. This enzyme activates the final step in the process of carbohydrate digestion resulting in the breakdown of disaccharides and oligosaccharides into absorbable glucose and inhibition of these hydrolytic enzymes may ameliorate the influx of glucose from the intestinal tract to blood vessels. According to our data, Laurel and Myrtle extract may be a potential inhibitor of carbohydrate-hydrolysing enzymes for a therapy of hypoglycaemia. The greatest inhibition of β-glucosidase, β-glucuronidase, β-galactosidase activity was shown by the use of Myrtle at a dose of 50 mg/kg; the reduction of these enzymes was as follows: 43.30%; 58.88%; and 65.61%, respectively. Treatment of rats with Laurel-100 caused the reduction of these enzymes for 27.66%; 28.15% and 39.74% compared to the control.

According to our results, it is clear that the application of Laurel and Myrtle extracts can be a way to reduce *Enterobacteriaceae*. These data may be an important strategy in humans and animals, since high faecal *Enterobacteriaceae* levels are associated with inflammatory bowel disease, immune imbalance and exacerbate the inflammatory status of the gut epithelium [38]. Significant reduction of *Enterobacteriaceae* was observed in all groups compared to control; treatment with Laurel-100 reduced number of colonies for 72% (*p* < 0.01), Myrtle-50 for 69.57% (*p* < 0.01) while Laurel-50 and Myrtle-100 show a reduction of 41.90% without significance (Figure 2c).

The consumption of Laurel and Myrtle extract can also Influence oxidative stress and its damaging power, in both a positive and a negative way. Hence, dietary strategies for reducing inflammation and oxidative stress could be an important approach for the prevention against chronic and degenerative diseases. It seems that a strategy to reduce *Enterobacteriaceae*-induced inflammation may be associated with antioxidant activity of polyphenols present in Laurel and Myrtle extract. The antioxidant effects of dietary polyphenols may be exerted in the digestive tract, because of their high concentration in the gut. According to our results (Figure 5), changes in the microbiota and their enzymes are partly related to the oxido-reduction status in rats after the administration of Myrtle at a dose of 50 mg/kg, as indicated by increased MDA activity in the renal homogenate as well as CAT activity in the liver homogenate. It is possible that increased metabolism also partially increases oxidative stress levels. The different antioxidant or pro-oxidative effect of Myrta extract can be explained in part by its polyphenolic constituents. In Myrtle extract, the main polyphenolic components are Myricetin-3-*O*-rham (36.68%), Myricetin-3-*O*-gallac (33.20%), Miricetin (14.48%) and 5-*O*-galloyl quinic Acid (7.96%). Myricetin can act as an antioxidant and as pro-oxidant, possesses six hydroxyl groups and thus has great potential for participating in oxidation and redox cycling as described in [39]. Its potential to act as a pro-oxidant due to its tendency to undergo autoxidation depending upon its environment resulting in changes in SOD and CAT activity. Myricetin, as a pro-oxidant, has the ability to increase the production of hydroxy radicals through reactions with Cu^2+^, Fe^2+^ or Fe^3+^-EDTA and hydrogen peroxide leading to DNA degradation.

Antioxidant capacity in renal and hepatic tissue was not changed when compared to the control which coincided with paper [40], where it has been shown that a longer time of plant extract intake is required to achieve the antioxidant capacity of the tissue. However, it should be noted that the possibility of adapting a healthy organism in maintaining the level of reactive radicals and antioxidant protection and that the effect of Laurel and Myrtle extracts, as antioxidants, was more clearly visible in diseases associated with oxidative stress.

It seems that polyphenols from Laurel extract have a better effect on the gut microbiota as compared to the Myrtle extract and that the increase in beneficial bacteria may act on polyphenols to increase their bioavailability. The release of functional phenolic constituents after microbial fermentation in the colon contributes to colon health through their antioxidative, anti-inflammatory and immunomodulatory effects. It is possible that Laurel extract increases the functional diversity of the microbiota, which is the goal of our further research. Through these prebiotic-effects, (poly)phenol-rich foods can attenuate metabolic and inflammatory diseases, increase host intestinal mucus production, induce the secretion of gut antimicrobial peptides, modulate hepatic bile acids, and gut immunoglobulins secretion [11,12,13,19,20,21,22,23]. According to [41,42,43,44,45,46,47], probiotic strains are involved in the scavenging of hydroxyl radicals and superoxide anions and produce antioxidants such as glutathione transferase, CAT, superoxide dismutase (SOD), GSH, folate, uric acid and vitamins C E, and β-carotene, which are absorbed and distributed in the organism. In this way SOD production intensifies the inherent cellular antioxidant defence, as it prevents the formation of the toxic superoxide anion catalysing its dismutation to hydrogen peroxide. The most commonly used strains are *Lactobacillus* and *Bifidobacterium*, which are reported to secrete SOD enzymes and metal-chelating and antioxidant molecules, and could protect the intestine, liver and kidney against diseases associated with oxidative stress, including even cancer [41,42,43,44,45]. In addition, exopolysaccharides which are released by probiotic bacteria potentially play a role in the oxidative stress reduction and stimulation of Nrf2 expression in the liver by *Lactobacillus* [44,47]. The antioxidative effect of probiotic bacteria, including *Lactobacillus rhamnosus* GG (LGG), *Lactobacillus paracasei* Fn032, *Lactobacillus plantarum* CAI6, *Lactobacillus casei*, *Lactobacillus plantarum* SC4, *Lactobacillus acidophilus*, *Lactobacillus bulgaricus DSMZ 20080* and *Bifidobacterium longum*, *Bifidobacterium lactis* and *Bifidobacterium breve* does not result from antioxidant capacity but from prevention of ROS generation [41,42,43,44,45,46,47]. *Lactobacillus* strains have been found to have higher total antioxidant activity than other examined strains, and their antioxidative activity is a strain-specific feature but can be related to the fermentation type of probiotic bacteria [44]. According to [48], LAB antioxidant activity depends on their concentration; LAB develop their protective properties when their titre was equal to 3 × 10^7^ cells/mL. It seems that the protective effect of Laurel against oxidative stress is based in part on its prebiotic properties through the restoration of the intestinal microbiota [49]. An additional explanation may be that probiotics can inhibit intestinal pathogens and reduce postprandial lipids which are involved in oxidative damage. In addition, *Lactococcus lactis* could be responsible for increased CAT activity in the liver after the ingestion of Myrtle at a dose of 50 mg/kg [50].

Taken together, our studies suggest that the ingestion of Laurel and Myrtle polyphenolic components could modulate the gut microbiota and their glycolytic activity leading to weight change but have a slow effect on liver and kidney antioxidative capacity.

## 4. Materials and Methods

### 4.1. Material

The air-dried Laurel leaves (*Laurus nobilis* L.) were purchased from Šafram Ltd. (Zagreb, Croatia), collected in November 2020 in the Lovran area (Istra, Croatia). Air-dried Myrtle leaves (*Myrtus communis* L.) collected in December 2020 in the area of Mljet (Dalmatia, Croatia) were purchased from Stella Mediterranea Ltd. (Klis, Croatia). The dried leaves were ground in an electric mill (GT11, Tefal, Rumily, France) and sieved through a 2-mm sieve before extraction.

### 4.2. Extraction

The extraction of phenolic compounds from ground Laurel and Myrtle samples was performed using 30% aqueous ethanol solution (*v*/*v*). The sample (10 g) was mixed with 100 mL of 30% ethanol and the extraction procedure was carried out in a water bath shaker at 60 °C for 20 min. The extraction process was repeated in several batches which were combined, and the combined extract was evaporated to a volume of 200 mL on a vacuum concentrator (Thermo Scientific Savant SPD2010 SpeedVac^®^ Concentrators, Ramsey, MN, USA), to reach the target total phenol concentration in the final extract of about 50 g GAE/L. The extract obtained is an aqueous extract; the ethanol was evaporated during the extract concentration process. The obtained extract was used for the determination of total phenols (TPC) and UPLC-MS^2^ analysis.

### 4.3. Determination of Total Phenolic Content in Laurel and Myrtle Extract

Phenolic compounds in Laurel and Myrtle extracts were estimated by spectrophotometric determination with a Folin–Ciocalteu method previously described by Oršolić and co-authors [11,51]. The aliquot (100 μL) of each sample extract was briefly mixed with 200 μL of Folin–Ciocalteu reagent and 2 mL of distilled water. After 3 min, 20% sodium carbonate solution (1 mL) was added to the mixture, left for 2 h at room temperature in the dark with occasional shaking, and the absorbance was measured at 765 nm (Shimadzu, Kyoto, Japan). The same procedure was repeated for the standard of gallic acid solutions. Total phenolic content was calculated according to the gallic acid standard calibration curve (y = 0.0035x, R^2^ = 0.9995) prepared from working standard solutions in concentration range from 50 to 500 mg/L in triplicate and was expressed on a fresh weight basis as mg of gallic acid equivalents (GAE)/100 g of the sample.

### 4.4. UPLC-MS^2^ Analysis

UPLC-MS^2^ analyses were performed on an Agilent 1290 series RRLC instrument (Agilent, Santa Clara, CA, USA) connected to an Agilent triple quadrupole mass spectrometer (6430) with an ESI ion source. Chromatographic separations were performed on an Agilent Zorbax Eclipse Plus C18 column (100 mm × 2.1 mm, 1.8 μm particle size). Ionization was performed by electrospray (ESI) in negative and positive modes (*m*/*z* 100 to 1000), and data were acquired in dynamic multiple reaction monitoring (dMRM) mode. The ionization source parameters were: positive/negative capillary voltage, 4000/3500 V, drying gas temperature of 300 °C with a flow rate of 11 L/h and a nebulizer pressure of 40 psi. High purity nitrogen (99.999%) was used as the inducing cone and collision gas, which was obtained from Messer (Osijek, Croatia). Analyses were performed according to a method previously described by Elez Garofulic et al. [52].

Identification was performed by comparing retention time and *m*/*z* values obtained from MS and MS2 with mass spectra of the corresponding standards. The identified polyphenols were quantified based on their peak areas and the calibration curves obtained with the corresponding standards. For the compounds for which there are no reference standards, identification was based on mass spectral data and the literature reports of characteristic fragmentation patterns for each compound, while quantification was performed through standard calibration curves for each available standard and as follows: kaempferol-3-rutinoside, kaempferol-3-*O*-hexoside, kaempferol-3-*O*-deoxyhexoside and kaempferol-3-*O*-pentoside according to the kaempferol-3-glucoside calibration curve, isorhamnetin-3-hexoside, quercetin-3-rhamnoside and quercetin-3-pentoside according to quercetin-3-glucoside, apigenin-6-C-(*O*-deoxyhexosyl)-hexoside corresponding to apigenin, luteolin glucoside corresponding to luteolin, epicatechin corresponding to catechin and 3,4-dihydroxybenzoic acid hexoside corresponding to the protocatechuic acid calibration curve.

### 4.5. Experimental Animals, Study Design and Organ Processing

Adult male Sprague-Dawley (220–240 g), three months old, obtained from Department of Animal Physiology, Faculty of Science, University of Zagreb, were used in the present study. The ethical committee (Faculty of Science, University of Zagreb, Croatia) approved present study (approval code: 251-58-10617-21-3). All the procedures complied with ethics guidelines such as the guidelines in force in Republic of Croatia (Law on the Welfare of Animals, NN135/06 and NN37/13), EU Directive 2010/63/EU for animal experiments (reference: OJEU 2010) and the Guide for the Care and Use of Laboratory Animals, DHHS Publ. # (NIH) 86-123. Animals were housed in plastic cages in a climate-controlled facility with a constant day–night cycle (light: 08.00–20.00 h) at a temperature of 25 ± 1 °C, and a relative humidity of 50 ± 10%. The animals were maintained on a formulated commercial pelleted diet and water was provided ad libitum. Food pellets were a certificated standard mice and rat diet 4RF21 (Mucedola, Italy; Batch No. 238603, shape 12 mm). Composition of standardized pellet rat feed included wheat, wheat straw, hazelnut skins, maize, soy bean dehulled, corn gluten feed, fishmeal, dicalcium phosphate, sodium chloride, whey powder, soybean oil, yeast; and contained 12% moisture, 18.5% protein, 3% fats, 6% crude fibres, 7% crude ash, E672 (vitamin A), E671 (vitamin E), E1 (Fe), E2 (I), E3 (Co), E4 (Cu), E5 (Mn), and E6 (Zn) [11,53].

After acclimatization, rats (*n* = 25) were divided into 5 groups: control group, Laurel extract at dose of 50 and 100 mg/kg, Myrtle extract at dose of 50 and 100 mg/kg [2,54]. Rats were treated by intragastric administration of Laurel and Myrtle extract once a day for 14 days. The dosing was adjusted according to the status of the rat’s weight on a daily basis. At 2 weeks after the treatment, the rats were anesthetized using a mixture of ketamine (Narketan^®^10, Vetoquinol AG, Belp Bern, Switzerland) at dose of 75 mg/kg with xylazine (Xylapana^®^ Vetoquinol Biowet Sp., Gorzow, Poland) at dose of 10 mg/kg. The livers and kidneys of each rat were dissected and weighed, and tissues were used to determine oxidative and anti-oxidative status by measuring the lipid peroxidation and glutathione levels, CAT activity and for antioxidative capacity analysis by methods such as ferric reducing antioxidant power (FRAP), ABTS•+ scavenging activity, and DPPH^•^ free radical scavenging activity. In addition, fresh faecal samples (weight 0.1 g) were collected from each rat for counting of the colonies of probiotic bacteria (Lactobacilli and Bifidobacteria) and *Enterobacteriaceae*, colonic microbiota enzyme activities (β-glucuronidase, β-glucosidase, β-galactosidase activity, and pH value). Experimental groups were additionally monitored for body weight change (body weight was recorded at the beginning and on termination of the study) as well as food and drink consumption.

### 4.6. Body Weight

Body weight was measured using a digital scale (Kern KB 2000-2N; d = 0.01–2000 g): (a) on the first day of the experiment, (b) every seven days for the duration of the experiment, and (c) on the day of sacrifice. The percentage of body weight change was calculated according to the formula:

$$\mathrm{Weight}\;\mathrm{change}\;\left(\%\right)\;=\;\left(\mathrm{final}\;\mathrm{weight}-\mathrm{initial}\;\mathrm{weight}\right)\;\times \;100/\left(\mathrm{final}\;\mathrm{weight}\right)$$

### 4.7. Evaluation of Food and Drink Consumption

Diets and the quantity of water were expressed on a daily basis. The consumption of food and drink was calculated using the difference between the initial and the final pellet weight and expressed as average consumed pellet weight per 24-h period per animal. The results were expressed daily in grams (g) or mL. Food efficiency ratio was calculated according to the formula:

Food efficiency ratio = weight gain (g)/food intake (g) × 100

### 4.8. Antioxidant Status of Tissues

The antioxidant capacities of rat liver and kidney tissues were examined for different antioxidant mechanisms, using the following methods described [19,20]: (i) Ferric reducing antioxidant power (FRAP) assay; (ii) ABTS assay (2,2′-azino-bis(3-ethylbenzothiazoline-6-sulfonic acid); (iii) DPPH• free radical scavenging assay (2,2-Diphenyl-1-(2,4,6-trinitrophenyl) hydrazyl and (iv) markers of tissue oxidative stress defence systems [Malondialdehyde (MDA), biomarkers for lipid peroxidation; glutathione level (GSH), intracellular and extracellular protective antioxidant; and catalase activity (CAT) a ubiquitous antioxidant enzyme that degrades hydrogen peroxide into water and oxygen].

Tissue supernatant samples were centrifuged at 20,000× *g* for 15 min at 4 °C and supernatant was used for analysis following protocols described in our previous paper [40,55].

Briefly, the **FRAP** reagent was prepared from 5 mL of a TPTZ solution (10 mM) in HCl (40 mM) and 5 mL of a FeCl_3_ solution (20 mM) mixed with 50 mL of an acetate buffer (0.3 M, pH = 3.6). A freshly prepared FRAP reagent (1.5 mL) was mixed with 200 µL of water and 50 µL of the tissue sample or as a blank standard sample with 50 µL water and incubated for 4 min at room temperature. The absorbance was measured at 595 nm with a Libro S22 spectrophotometer (Biochrom Ltd., Cambridge, UK) and the ferric reducing ability of the tissue homogenate was calculated according to the standard curve and expressed as nmol Fe^2+^ per mg of protein in samples. The analyses were performed in triplicate.

In **ABTS assay**, 20 μL of the tissue supernatant was mixed with 2 mL of an ABTS•+ solution (7 mM ABTS•+ solution with a freshly prepared 140 mM potassium peroxydisulfate solution mixed in equal proportions), and incubated for 6 min. After incubation, the absorbance was measured at a wavelength of 734 nm with a Libro S22 spectrophotometer (Biochrom). The results of triplicate samples are expressed as nmol Trolox equivalents per mg of protein in the tissue homogenate.

The **DPPH free radical assay** was carried out in a 96-well microplate using the method previously described [36] with modifications. Briefly, 10 µL of extract was added to 290 µL of 0.08 mg/mL ethanolic DPPH solution. The alcoholic DPPH solution changes from deep purple to yellow during the reaction. The plate was incubated for 60 min in the dark at room temperature and the absorbance was recorded at 517 nm using a Libro S22 spectrophotometer (Biochrom Ltd., Cambridge, UK).

Biomarkers for **lipid peroxidation** (**MDA**) in tissues were determined by measuring the content of TBARS using a method described by our previous paper [38,39]. Briefly, after centrifugation supernatant of the homogenized tissue (200 µL) was mixed with 200 μL of 8.1% sodium dodecyl sulphate (SDS), 1.5 mL of 20% acetic acid (pH = 3.5), and 1.5 mL of 0.81% thiobarbituric acid, and incubated at 95 °C for 60 min. Absorbance was measured at 532 and 600 nm with a Libro S22 spectrophotometer (Biochrom) using the formula:

A_total_ = A_532_ nm − A_600_ nm

The concentration of lipid peroxides was expressed as nmol MDA/mg protein.

**The reduced glutathione (GSH) assay** was determined by mixing 40 μL of 10 mM DTNB (Ellman’s Reagent) with 20 μL of the tissue supernatant pretreated with 40 µL of 0.035 M HCl and incubated for 10 min. DTNB reacts with GSH to form the chromospheres, 5-thionitrobenzoic acid (TNB) and GS-TNB; the absorbance was measured at 412 nm and expressed as µM/mg proteins.

**CAT activity** was prepared by mixing 33 mM H_2_O_2_ in 50 mM phosphate buffer, pH = 7.0. This reaction mixture (900 µL) was mixed with the supernatant of the tissue homogenate (100 µL) and measured by the extinction coefficient of H_2_O_2_ (ε = 39.4 m/Mcm); the specific activity was expressed as U/mg protein. CAT activity was estimated by the decrease in absorbance of H_2_O_2_ at 240 nm using the Libro S22 spectrophotometer (Biochrom).

**The concentration of carbonylated proteins** in the liver and kidney tissue samples was described in paper [36]. The method is based on the reaction of carbonyl groups of the protein chain with 2,4-dinitrophenylhydrazine (DNPH) in an acidic medium to form 2,4-dinitrophenylhydrazone. First, 200 μL of supernatant sample and 300 μL of 10 mM DNPH dissolved in 2 M HCl were added to each tube and the samples were incubated at room temperature for one hour with occasional stirring (Vortex, Genius 3, IKA, Wilmington, NC, USA). After incubation, the proteins were precipitated with 500 μL of 20% trichloroacetic acid (TCA) at −20 °C for five minutes, followed by centrifugation of the samples for 10 min at 12,000 rmp and 4 °C. After centrifugation, the precipitate was resuspended in 1 mL of a 1:1 solution of ethanol and ethyl acetate and centrifuged for 10 min at 10,000 rmp and 4 °C. The precipitate washing procedure was repeated three times to wash the unbound 2,4-dinitrophenylhydrazine. The precipitate was then dissolved in 1 mL of 6 M guanidine hydrochloride by vortexing and incubation at 37 °C for five minutes. The concentration of carbonylated proteins was determined on a spectrophotometer by measuring the absorbance at a wavelength of 370 nm (Libra S22, Biochrom Ltd., Cambridge, UK), and 2 M HCl was used as a blank. The concentration of carbonylated proteins was calculated using the molar extinction coefficient (ε = 0.011 µ/M) according to the following formula:

c = (A_sample − A_ (blanks))/ε

The concentration of carbonylated proteins was expressed as nmol/mg protein.

### 4.9. Preparation of Nutrient Media

Solid media, i.e., selective media with inhibitory activity on other microorganisms and broths, were used for isolation and determination of microorganisms. The process of preparation of nutrient media was performed according to the manufacturer’s instructions and they were sterilized in an autoclave. After autoclaving, the substrates were cooled in a water bath 47 °C–50 °C and poured into sterile Petri dishes. The substrates prepared in this way were kept from drying out and protected from light in a refrigerator at a temperature of 5 °C.

### 4.10. Intestinal Sampling

The colon was isolated immediately after the proper killing of the animal. Following aseptic rules, 100 mg of colon contents were sampled for measurement of glycolytic activity of intestinal microbiota enzymes, and for the isolation of probiotic cultures. To isolate probiotic bacteria and Enterobacteria, the sampled colon contents were resuspended in thioglycolate at a ratio of 1:10 (*w*/*v*).

### 4.11. Determination of the Number of Probiotic Bacteria

The total number of Lactobacilli and Bifidobacteria were enumerated using nutrient agar and incubated at a temperature of 37 °C in accordance with ISO standards (Lactobacilli according to the ISO 20128: 2006 standard and Bifidobacteria according to the ISO 29981: 2010 standard). For the determination of Lactobacilli, 0.1 mL of the sample was inoculated on MRS agar, and incubation was performed under microaerophilic conditions using Oxoid™ CampyGen™ 2.5 L Sachet (Thermo Fisher Scientific Inc., Milan, Italy) for 72 ± 2 h. *Bifidobacterium* were inoculated on TOS agar (0.1 mL of samples) and incubation was performed under anaerobic conditions using Oxoid™ AnaeroGen™ 2.5 L Sachet (Thermo Fisher Scientific Inc., Milan, Italy) for 72 ± 2 h. The total number of isolated microorganisms is expressed as the logarithm of the number of cells (log10 CFU mL^−1^). Formula for calculating the number of cells:

CFU = (number of colonies grown)/(sample volume used) × reciprocal value of decimal dilution

In plates with *Lactobacilli* growth, 5 typical colonies were isolated on CASO agar (37 °C/24 ± 2 h) and biochemical confirmation was made. Biochemical confirmation was performed using API 50 CHL (BioMérieux, Marcy-l-Etoile, France), which is commercially available, and the procedure was done according to the manufacturer’s instructions.

### 4.12. Determination of the Presence and Enumeration of Bacteria from the Family Enterobacteriaceae

The procedure for proving the presence and enumeration of viable bacteria from the *Enterobacteriaceae* family by inoculating a sample on a selective violet-red brilliant agar (VRBG agar) was performed according to the HRN EN ISO 21528-2: 2017 standard. The sample (1 mL) was inoculated into two sterile Petri dishes. Approximately 10 mL of dissolved VRBG agar was added to each Petri dish with the inoculated sample. The closed Petri plate was carefully rotated to mix the inoculated contents with the agar. After the agar solidified on the surface, another 15 mL of agar was added. Inoculated Petri dishes were incubated with the lid turned down at 37 ± 1 °C for 24 ± 2 h. After incubation, typical rose colonies of Enterobacteria that are red or pink were counted. On plates in which there was growth of Enterobacteria, 5 typical colonies were isolated on nutrient agar (37 °C/24 ± 2 h) and biochemical confirmation was made by oxidase assay (Merck). Characteristic colonies on VRBG were Gram-stained and tested for their oxidase reaction; Gram-negative, oxidase-negative bacteria were presumptively identified as *Enterobacteriaceae*.

### 4.13. Determination of Glycolytic Enzymatic Activity of Bacteria in a Sample Isolated from the Intestine

The collected faeces and colon contents were also used for the determination of β-galactosidase, β-glucuronidase and β-glucosidase enzyme activities. Glycolytic activity of bacteria in the contents of the colon was measured by the rate of release of p- and o-nitrophenols from their nitrophenylglucosides according to the method of Juśkiewicz et al. [33]. The following substrates were used for enzymes at a concentration of 5 mM: for β-glucuronidase substrate p-nitrophenyl-β-D-glucuronide, for substrate p-nitrophenyl-β-D-glucopyranoside and for β-galactosidase substrate o -β-D-galactopyranoside (Sigma-Aldrich, Darmstadt, Germany), and were prepared by being dissolved in 100 mL of freshly prepared 100 mM phosphate buffer (pH, 7.0). The reaction mixture contained 0.3 mL of substrate solution and 0.2 mL of 1:10 (*v*/*v*) supernatant colon content, which was further diluted in 100 mM phosphate buffer. Incubation at 37 °C/10 min/anaerobically followed. After incubation, 2.5 mL of cold sodium carbonate (0.25 M) was added to stop the reaction, and the absorbance of p-nitrophenol at λ = 400 nm and o-nitrophenol at λ = 420 nm was measured on a spectrophotometer (Biochrome, Cambridge, UK). Enzyme activity (U/g c.c.) was calculated according to the formula: U = [[(A: 0.04401): 10]: 0.02]: 139.11. Where A is the absorbance; 0.4401—slope of the direction (determined from the calibration diagram for p-nitrophenol (PNP) and o-nitrophenol (OPN)); 10—incubation time in minutes; 0.02—amount of sample expressed in grams (in 0.2 mL of supernatant): Mr (PNP/ONP) = 139.11 g/mol. The assay was performed in triplicates. The enzyme inhibitory rates of Laurel and Myrtle extracts were calculated according to the following equation:

Inhibitory rate (%) = [(A control − A sample)/A control] × 100

### 4.14. Statistical Analyses

The data were presented as mean ± standard error (SE) values. All data were analysed using the Kruskal-Wallis nonparametric test. Further analysis of the differences between the groups was made with multiple comparisons of mean ranks for all groups. Statistical analyses were performed using STATISTICA 14software (StatSoft, Tulsa, OK, USA). The data were considered significant at *p* < 0.05.

## 5. Conclusions

According to the results, the phenolic components of Laurel extract have a better effect on the intestinal microbiota and their metabolic activity than Myrtle extract. The application of high doses of Laurel extract increases the number of probiotic bacteria by reducing the growth of pathogenic bacteria and thus ensures better health status of animals. Furthermore, these studies confirm the beneficial effect of Laurel and Myrtle extract in combating pathogenic Gram-negative *Enterobacteriaceae* strains that includes bacteria with a wide range of healthcare-related infections. Therefore, low toxicity of flavonoids present in Laurel extract and Myrtle at a lower dose may be a good approach to maintaining animal and human health but also a good strategy for treatment against infectious diseases in the future. This two-way interaction of polyphenols from Laurel and Myrtle extract and intestinal microbiota affects metabolic pathways leading to health benefits and prevention of potential disease development.

## Figures and Tables

**Table 1:** Main components (%) in water extract of *Laurus nobilis* L. and *Myrtus communis* L. detected by UPLC-MS^2^ analysis.

	Polyphenol (%)		MS/MS Fragments	
Compound	*Laurus nobilis* L.	*Myrtus communis* L.	Product Ion *m*/*z*	Fragment Ion *m*/*z*
3,4 dihydroxybenzoic acid hexoside	0.87	-	317	155
5-*O*-galloylquinic acid	-	7.96	343	191
Apigenin	12.63	-	271	153
Apigenin-6-C-(*O*-deoxyhexosyl)-hexoside	0.71	-	579	459
Caffeic acid	19.31	1.81	179	135
Catechin	4.09	0.05	291	139
Chlorogenic acid	0.14	-	353	191
Digalloylquinic acid	-	0.79	495	343
Ellagic acid	-	0.03	301	257
Epicatechin	6.77	0.05	291	139
Epicatechingallate	0.58	0.02	442.9	273
Epigallocatechingallate	0.39	-	459	139
Ferulic acid	1.12	-	193	134
Gallic acid	0.51	-	169	125
Kaempferol	-	-	287	153
Kaempferol deoxyhexoside	0.07	-	433	286
Kaempferol hexoside	5.24	-	449	287
Kaempferol pentoside	1.61	-	419	287
Kaempferol-3-rutinoside	0.62	-	595	287
lsorhamnetin hexoside	4.66	-	479	317
Luteolin	2.32	1.11	287	153
Luteolin glucoside	0.33	2.63	449	287
Myricetin	3.45	14.48	319	273
Myricetin-3-*O*-arabinoside	-	0.05	452	319
Myricetin-3-*O*-galactoside	-	33.20	481	319
Myricetin-3-*O*-rhamnoside	-	36.68	465	319
*p*-Coumaric acid	0.95	-	163	119
Procyanidin trimer	2.77	-	865	713
Protocatechuic acid	1.69	-	153	109
Quercetin	-	-		
Quercetin-3-glucoside	10.74	0.85	465	303.1
Quercetin-3-pentoside	4.04	-	435	303
Quercetin-3-rhamnoside	1.15	-	449	303
Quercetin-3-rutinoside	12.24	-	611	303
Quercitrin	-	0.25		
Rosmarinic acid	0.84	-	359,08	161
Syringic acid	0.17	-	197	182
**Total (mg/mL)**	**51.36**	**48.64**		

**Table 2:** Weight gain, food intake, food efficiency ratio (FER) in Laurel and Myrtle extract fed rats.

Treatments ^a^	Daily Weight Gain (g)	Food Intake (g/Daily)	Food Efficiency Ratio (FER) ^b^
Laurel-100	2.85 ± 0.55 ^■^	11.23 ± 0.50	0.25 ± 0.03 ^■^
Laurel-50	1.44 ± 0.22	14.67 ± 0.09	0.10 ± 0.01
Myrtle-100	0.86 ± 0.14	12.76 ± 0.11	0.07 ± 0.03
Myrtle-50	3.59 ± 0.42 ^■■^	11.20 ± 0.4	0.32 ± 0.02 ^■■^
Control	1.53 ± 0.24	13.31± 0.07	0.12 ± 0.01

^a^ Male rats (*n* = 5) were administered with Laurel and Myrtle extract *ig* at a dose of 50 and 100 mg/kg once a day for 14 days. The control group was treated with *ig* saline. The results are expressed as the mean value of each experimental group ± SE of the mean of two different observations. ^b^ Food efficiency ratio: weight gain (g)/food intake (g/daily). ^■^ Significantly different in relation to Myrtle-100 (^■^ *p* < 0.05; ^■■^ *p* < 0.01).

## Data Availability

Not applicable.

## Supplementary Materials

The following supporting information can be downloaded. Table S1: The effect of Laurel and Myrtle extract on the pH value of the intestinal contents of the colon in rat.

## Sample Availability

Samples of the compounds are available from the authors.

## References

[1] Alipour G., Dashti S., Hosseinzadeh H. (2014). Review of pharmacological effects of *Myrtus communis* L. and its active constituents. Phytother. Res..

[2] Hennia A., Miguel M., Nemmiche S. (2018). Antioxidant Activity of *Myrtus communis* L. and Myrtus nivellei Batt. & Trab. Extracts: A Brief Review. Medicines.

[3] Conforti F., Statti G., Uzunov D., Menichini F. (2006). Comparative Chemical Composition and Antioxidant Activities of Wild and Cultivated *Laurus nobilis* L. Leaves and *Foeniculum vulgare* subsp. piperitum (Ucria) Coutinho Seeds. Biol. Pharm. Bull..

[4] Gazwi H.S.S., Yassien E.E., Hassan H.M. (2020). Mitigation of lead neurotoxicity by the ethanolic extract of Laurus leaf in rats. Ecotoxicol. Environ. Saf..

[5] Serçe S., Ekbiç E., Suda J., Gündüz K., Kiyga Y. (2010). Karyological features of wild and cultivated forms of myrtle (*Myrtus communis*, Myrtaceae). Genet. Mol. Res..

[6] Alejo-Armijo A., Altarejos J., Salido S. (2017). Phytochemicals and Biological Activities of Laurel Tree (*Laurus nobilis*). Nat. Prod. Commun..

[7] Gasparyan G., Tiratsuyan S., Kazaryan S., Vardapetyan H. (2015). Effect of *Laurus nobilis* extract on the functioning of liver against CCl4 induced toxicity. J. Exp. Biol. Agric. Sci..

[8] Amensour M., Sendra E., Abrini J., Bouhdid S., Pérez-Alvarez J.A., Fernández-López J. (2009). Total Phenolic Content and Antioxidant Activity of Myrtle (*Myrtus communis*) Extracts. Nat. Prod. Commun..

[9] Bento-Silva A., Koistinen V.M., Mena P., Bronze M.R., Hanhineva K., Sahlstrøm S., Kitryte V., Moco S., Aura A.-M. (2019). Factors affecting intake, metabolism and health benefits of phenolic acids: Do we understand individual variability?. Eur. J. Nutr..

[10] Singh M., Thrimawithana T., Shukla R., Adhikari B. (2020). Managing Obesity through Natural Polyphenols: A Review. Future Foods.

[11] Oršolić N., Landeka Jurčević I., Đikić D., Rogić D., Odeh D., Balta V., Perak Junaković E., Terzić S., Jutrić D. (2019). Effect of Propolis on Diet-Induced Hyperlipidemia and Atherogenic Indices in Mice. Antioxidants.

[12] Mithul A.S., Wichienchot S., Tsao R., Ramakrishnan S., Chakkaravarthi S. (2021). Role of dietary polyphenols on gut microbiota, their metabolites and health benefits. Food Res. Int..

[13] Oršolić N., Jazvinšćak Jembrek M., Terzić S. (2017). Honey and quercetin reduce ochratoxin A-induced DNA damage in the liver and the kidney through the modulation of intestinal microflora. Food Agric. Immunol..

[14] Abdul-Lateef Ali N., Baqur Sahib Al-Shuhaib M. (2021). Highly effective dietary inclusion of laurel (*Laurus nobilis*) leaves on productive traits of broiler chickens. Acta Sci. Anim. Sci..

[15] Abd El-Hack M.E., Abdelnour S.A., Taha A.E., Khafaga A.F., Arif M., Ayasan T., Swelum A.A., Abukhalil M.H., Alkahtani S., Aleya L. (2019). Herbs as thermoregulatory agents in poultry: An overview. Sci. Total Environ..

[16] Bayliak M.M., Burdyliuk N.I., Lushchak V.I. (2016). Effects of pH on antioxidant and prooxidant properties of common medicinal herbs. Open Life Sci..

[17] Oršolić N., Kunštić M., Kukolj M., Odeh D., Ančić D. (2020). Natural phenolic acid, product of honey bee, in control of oxidative stress, peritoneal angiogenesis and tumor growth in mice. Molecules.

[18] Mahfuz S., Piao X.S. (2019). Application of Moringa (*Moringa oleifera*) as Natural Feed Supplement in Poultry Diets. Animals.

[19] Million M., Angelakis E., Paul M., Armougom F., Leibovici L., Raoult D. (2012). Comparative meta-analysis of the effect of *Lactobacillus* species on weight gain in humans and animals. Microb. Pathog..

[20] Million M., Maraninchi M., Henry M., Armougom F., Richet H., Carrieri P., Valero R., Raccah D., Vialettes B., Raoult D. (2012). Obesity-associated gut microbiota is enriched in *Lactobacillus reuteri* and depleted in Bifidobacterium animalis and *Methanobrevibacter smithii*. Int. J. Obes..

[21] Schellekens H., Torres-Fuentes C., van de Wouw M., Long-Smith C.M., Mitchell A., Strain C., Berding K., Bastiaanssen T.F.S., Rea K., Golubeva A.V. (2021). *Bifidobacterium longum *counters the effects of obesity: Partial successful translation from rodent to human. EBioMedicine.

[22] Zhang C., Yu Z., Zhao J., Zhang H., Zhai Q., Chen W. (2019). Colonization and probiotic function of *Bifidobacterium longum*. J. Funct. Foods.

[23] Yin Y.N., Yu Q.F., Fu N., Liu X.W., Lu F.G. (2010). Effects of four Bifidobacteria on obesity in high-fat diet induced rats. World J. Gastroenterol..

[24] Damgaard T.D., Otte J.A.H., Meinert L., Jensen K., Lametsch R. (2014). Antioxidant capacity of hydrolyzed porcine tissues. Food Sci. Nutr..

[25] Su H.-M., Feng L.-N., Zheng H.-D., Chen W. (2016). Myricetin protects against diet-induced obesity and ameliorates oxidative stress in C57BL/6 mice. J. Zhejiang Univ. Sci. B.

[26] Quercia S., Candela M., Giuliani C., Turroni S., Luiselli D., Rampelli S., Brigidi P., Franceschi C., Bacalini M.G., Garagnani P. (2014). From lifetime to evolution: Timescales of human gut microbiota adaptation. Front. Microbiol..

[27] Dashnyam P., Mudududdla R., Hsieh T.-J., Lin T.-C., Lin H.-Y., Chen P.-Y., Hsu C.-Y., Lin C.-H. (2018). β-Glucuronidases of opportunistic bacteria are the major contributors to xenobiotic-induced toxicity in the gut. Sci. Rep..

[28] Gloux K., Berteau O., El Oumami H., Beguet F., Leclerc M., Dore J. (2010). A metagenomic -glucuronidase uncovers a core adaptive function of the human intestinal microbiome. Proc. Natl. Acad. Sci. USA.

[29] Pellock S.J., Redinbo M.R. (2017). Glucuronides in the gut: Sugar-driven symbioses between microbe and host. J. Biol. Chem..

[30] Oyetayo V.O., Adetuyi F.C., Akinyosoye F.A. (2003). Safety and protective effect of *Lactobacillus acidophilus *and *Lactobacillus casei* used as probiotic agent in vivo. Afr. J. Biotechnol..

[31] Drissi F., Merhej V., Angelakis E., El Kaoutari A., Carrière F., Henrissat B., Raoult D. (2014). Comparative genomics analysis of *Lactobacillus* species associated with weight gain or weight protection. Nutr. Diabetes.

[32] Flores R., Shi J., Gail M.H., Gajer P., Ravel J., Goedert J.J. (2012). Association of Fecal Microbial Diversity and Taxonomy with Selected Enzymatic Functions. PLoS ONE.

[33] Juśkiewicz J., Zduńczyk Z., Wróblewska M., Gulewicz K. (2003). Influence of oligosaccharide extracts from pea and lupin seeds on caecal fermentation in rats. J. Anim. Feed Sci..

[34] Kim D., Beck B.R., Heo S.B., Kim J., Kim H.D., Lee S.M., Kim Y., Oh S.Y., Lee K., Do H. (2013). Lactococcus lactis BFE920 activates the innate immune system of olive flounder (*Paralichthys olivaceus*), resulting in protection against Streptococcus iniae infection and enhancing feed efficiency and weight gain in large-scale field studies. Fish Shellfish Immunol..

[35] Nguyen T.L., Chun W.K., Kim A., Kim N., Roh H.J., Lee Y., Yi M., Kim S., Park C.I., Kim D.H. (2018). Dietary Probiotic Effect of Lactococcus lactis WFLU12 on Low-Molecular-Weight Metabolites and Growth of Olive Flounder (*Paralichythys olivaceus*). Front. Microbiol..

[36] McIntosh F.M., Maison N., Holtrop G., Young P., Stevens V.J., Ince J., Johnstone A.M., Lobley G.E., Flint H.J., Louis P. (2012). Phylogenetic distribution of genes encoding β-glucuronidase activity in human colonic bacteria and the impact of diet on faecal glycosidase activities. Environ. Microbiol..

[37] Michlmayr H., Kneifel W. (2013). β-Glucosidase activities of lactic acid bacteria: Mechanisms, impact on fermented food and human health. FEMS Microbiol. Lett..

[38] O’Connor K., Morrissette M., Strandwitz P., Ghiglieri M., Caboni M., Liu H., Khoo C., D’Onofrio A., Lewis K. (2019). Cranberry extracts promote growth of Bacteroidaceae and decrease abundance of Enterobacteriaceae in a human gut simulator model. PLoS ONE.

[39] Sadžak A., Vlašić I., Kralj Z., Batarelo M., Oršolić N., Jazvinšćak Jembrek M., Kušen I., Šegota S. (2021). Neurotoxic Effect of Flavonol Myricetin in the Presence of Excess Copper. Molecules.

[40] Balta V., Đikić D., Crnić I., Odeh D., Oršolić N., Kmetič I., Murati T., Dragović Uzelac V., Landeka Jurčević I. (2020). The effects of four-week intake of blackthorn flower extract on mice tissue antioxidant status and phenolic content. Pol. J. Food Nutr. Sci..

[41] Spyropoulos B.G., Misiakos E.P., Fotiadis C., Stoidis C.N. (2011). Antioxidant properties of probiotics and their protective effects in the pathogenesis of radiation-induced enteritis and colitis. Dig. Dis. Sci..

[42] Kanmani P., Satish Kumar R., Yuvaraj N., Paari K.A., Pattukumar V., Arul V. (2013). Probiotics and its functionally valuable products—A review. Crit. Rev. Food Sci. Nutr..

[43] Nowak A., Paliwoda A., Błasiak J. (2019). Anti-proliferative, pro-apoptotic and anti-oxidative activity of *Lactobacillus* and *Bifidobacterium* strains: A review of mechanisms and therapeutic perspectives. Crit. Rev. Food Sci. Nutr..

[44] Hoffmann A., Kleniewska P., Pawliczak R. (2021). Antioxidative activity of probiotics. Arch. Med. Sci..

[45] Kim H., Kim J.S., Kim Y.G., Jeong Y., Kim J.-E., Paek N.-S., Kang C.-H. (2020). Antioxidant and Probiotic Properties of Lactobacilli and Bifidobacteria of Human Origins. Biotechnol. Bioproc. Eng..

[46] Ayyanna R., Ankaiah D., Arul V. (2018). Anti-inflammatory and antioxidant properties of probiotic bacterium Lactobacillus mucosae AN1 and *Lactobacillus fermentum *SNR1 in wistar albino rats. Front. Microbiol..

[47] Kodali V.P., Sen R. (2008). Antioxidant and free radical scavenging activities of an exopolysaccharide from a probiotic bacterium. Biotechnol. J..

[48] Choi S.S., Kim Y., Han K.S., You S., Oh S., Kim S.H. (2006). Effects of Lactobacillus strains on cancer cell proliferation and oxidative stress in vitro. Lett. Appl. Microbiol..

[49] Amaretti A., di Nunzio M., Pompei A., Raimondi S., Rossi M., Bordoni A. (2013). Antioxidant properties of potentially probiotic bacteria: In vitro and in vivo activities. Appl. Microbiol. Biotechnol..

[50] De Moreno de le Blanc A., LeBlanc J.G., Perdigón G., Miyoshi A., Langella P., Azevedo V., Sesma F. (2008). Oral administration of a catalase-producing Lactococcus lactis can prevent a chemically induced colon cancer in mice. J. Med. Microbiol..

[51] Oršolić N., Benković V., Horvat-Knežević A., Kopjar N., Kosalec I., Bakmaz M., Mihaljević Ž., Bendelja K., Bašić I. (2007). Assessment by survival analysis of the radioprotective properties of propolis and its polyphenolic compounds. Biol. Pharm. Bull..

[52] Elez Garofulić I., Zorić Z., Pedisić S., Brnčić M., Dragović-Uzelac V. (2019). UPLC-MS2 Profiling of Blackthorn Flower Polyphenols Isolated by Ultrasound-Assisted Extraction. J. Food Sci..

[53] Ilić I., Oršolić N., Rođak E., Odeh D., Lovrić M., Mujkić R., Delaš Aždajić M., Grgić A., Tolušić Levak M., Vargek M. (2020). The effect of high-fat diet and 13-cis retinoic acid application on lipid profile, glycemic response and oxidative stress in female Lewis rats. PLoS ONE.

[54] Qnais E.Y., Abdulla F.A., Kaddumi E.G., Abdalla S.S. (2012). Antidiarrheal activity of *Laurus nobilis* L. leaf extract in rats. J. Med. Food.

[55] Oršolić N., Nemrava J., Jeleč Ž., Kukolj M., Odeh D., Terzić S., Fureš R., Bagatin T., Bagatin D. (2018). The Beneficial Effect of Proanthocyanidins and Icariin on Biochemical Markers of Bone Turnover in Rats. Int. J. Mol. Sci..

