# Supplementary material for: Beneficial Effects of Laurel (Laurus nobilis L.) and Myrtle (Myrtus communis L.) Extract on Rat Health
Source: Molecules. 2022 Jan 17;27(2):581. doi: 10.3390/molecules27020581 (PMC8778765; doi:10.3390/molecules27020581)
Supplement: Supplementary file 1 [file molecules-27-00581-s001.zip › molecules-1513274-supplementary.pdf]

Table S1. The effect of Laurel and Myrtle extract on the pH value of the intestinal contents of the colon in rat.

Tretments[a]	pH value (Mean ± SE)	Minimum	Maximum
Laurel - 100	7.16 ± 0.12	6.87	7.61
Laurel - 50	7.01 ± 0.10	6.72	7.24
Myrtle - 100	7.16 ± 0.15	6.81	7.62
Myrtle - 50	7.00 ± 0.14	6.7	7.38
Control	7.24 ± 0.20	6.84	7.89

[a]Male rats (n=5) were administered Laurel and Myrtle extracts *ig* at a dose of 50 and 100 mg/kg once a day for 14 days. The control group was treated with *ig* saline. The results are expressed as the mean value of each experimental group ± SE of the mean of two different observations.
